# Nodular goiter and hyperplastic lesion of the thyroid share common deregulated expression profiles

**DOI:** 10.1186/1471-2164-15-S2-P70

**Published:** 2014-04-02

**Authors:** Ohoud Subhi, Nadia Baqtian, Manar Ata, Sajjad Karim, Khalid Al-Ghamdi, Osman Abdel Al-Hamour, Mohammed Hussein Al-Qahtani, Hans-Juergen Schulten, Jaudah Al-Maghrabi

**Affiliations:** 1 Center of Excellence in Genomic Medicine Research, King Abdulaziz University, Jeddah, Kingdom of Saudi Arabia; 2 Department of Surgery, Faculty of Medicine, King Abdulaziz University, Jeddah, Kingdom of Saudi Arabia; 3 Department of Surgery, King Faisal Specialist Hospital and Research Center, Jeddah, Kingdom of Saudi Arabia; 4 Department of Pathology, Faculty of Medicine, King Abdulaziz University, Jeddah, Kingdom of Saudi Arabia; 5 Department of Pathology, King Faisal Specialist Hospital and Research Center, Jeddah, Kingdom of Saudi Arabia

## Background

Proliferative thyroid lesions including nodular goiter and hyperplastic lesion are very common in the Middle East and North African (MENA) region [1]. Hyperplastic lesions are also regarded as a subcategory of goiter. High-density expression profiles in these benign thyroid lesions are not surveyed in detail [2]. In an effort to establish gene expression profiles that distinguish both lesions from each other and from normal thyroid (TN) tissue, we employed state-of-the-art oligonucleotide microarray technology.

## Materials and methods

Whole transcript expression profiles were generated in 17 goiters, 14 hyperplastic lesions and 7 TN samples utilizing Affymetrix HuGene 1.0 ST arrays. We used the default analysis method for generating a threshold of significance for differential expression (p-value with a false discovery rate ≤ 0.05 and a fold change > 2). Partek Genomics Suite and Ingenuity Pathway Analysis software packages were utilized to interpret data sets.

## Results

Expression profiles of goiters and hyperplastic lesions were highly related and no transcripts were differentially expressed between these two thyroid lesions under the given statistical threshold values. However, more than 10000 genes were differentially expressed between goiters, as well as hyperplastic lesions, and TN samples. The most differentially expressed transcripts were in fact downregulated in both thyroid lesions in comparison to TN samples and include genes like olfactory receptor, family 6, subfamily N (OR6N2), glial cells missing homolog 1, Drosophila (GCM1), family with sequence similarity 138, member B (FAM138B), prostate-specific P704P mRNA (P704P), and olfactory receptor, family 5, subfamily H, member 14 (OR5H14). The most upregulated transcripts in goiters and hyperplastic lesions vs TN samples include genes as cytochrome c oxidase assembly protein COX15 homolog (COX15), dyskeratosis congenita 1, dyskerin (DKC1), and DnaJ (Hsp40) homolog, subfamily A, member 2 (DNAJA2). Networks which were most deregulated in goiter and hyperplastic lesion in comparison to TN tissue share similar functions although certain pathways seem to be differentially affected in both thyroid lesions.

## Conclusions

Our study indicates that goiter and hyperplastic lesion share common deregulated expression profiles in comparison to TN tissue. As a certain number of goiters and hyperplastic lesions bear the capacity to develop to thyroid neoplasms, knowledge of deregulated genes in these lesions may help to identify patients which are at elevated risk for developing thyroid carcinoma. Further studies have to reveal which expression signatures in these benign thyroid lesions are in common with malignant cases.

*This study was supported by King Abdulaziz City for Science and Technology* (*KACST*) *grants 09-BIO707-03 and 09-BIO820-03.*
